# Wrist Function Test and Its Use to Assess Treatment Efficacy in Ischemic Stroke Survivors—A Pilot Study

**DOI:** 10.3390/diagnostics15070840

**Published:** 2025-03-25

**Authors:** Dmitry Skvortsov, Danila Lobunko, Anna Lobunko, Nina Belonovskaya, Galina Ivanova

**Affiliations:** 1 Center for Brain and Neurotechnology, Moscow 117513, Russia; 2 Research and Clinical Centre of Moscow, Moscow 107031, Russia

**Keywords:** stroke, rehabilitation, functional electrical stimulation, upper extremities, motor functions, wrist joint

## Abstract

Stroke is a major health issue causing high mortality and disability rates. Around 80% of stroke survivors experience impaired upper limb function, which is typically evaluated clinically. Functional electrical stimulation (FES) may help in motor function recovery. This study aimed to create an objective test of wrist flexion and extension functions and to assess the use of such a test in evaluating the efficacy of conventional rehabilitation therapy, alone and in combination with FES, in patients with a paretic upper limb. **Background/Objectives:** A total of 55 subjects were involved: 15 healthy volunteers and 40 post-stroke patients. The patients were split into two groups: one receiving only conventional rehabilitation (control group) and another receiving both conventional and FES therapies (FES group). **Methods:** Inertial sensors measured wrist flexion and extension parameters before and after the study treatment. **Results:** Both groups showed improvement based on the ARAT and FMA-UE scales. Normative values were established in the healthy group, revealing interhemispheric asymmetry. The wrist motion amplitudes and phases in both patient groups differed significantly from the healthy group. Initially, the paretic side had a 40-degree reduction compared to healthy subjects, while the non-paretic side showed a 10–17-degree decrease. After treatment, the FES group demonstrated a 4–10-degree increase in the wrist motion amplitude on the paretic side. The phase parameters did not change significantly in either group. **Conclusions**: The developed wrist flexion–extension test was shown to be objective and sensitive. The FES treatment improved the movement amplitude, although it did not alter the temporal structure of motion in both patient groups.

## 1. Introduction

Stroke is associated with high mortality and disability rates and, therefore, presents a serious medical and social problem. The WHO predicts an increase in the number of stroke cases [1]. Stroke can cause motor, sensory, visual, affective, cognitive, and speech disorders. Functional disorders of the upper extremities persist in 80% of ischemic stroke survivors despite administered rehabilitation [2].

Although rehabilitation methods help to improve motor functions [3], the efficacy of most common protocols is often low [4]. In recent years, researchers’ attention has been drawn to functional electrical stimulation (FES). This rehabilitation technique is based on sending short electrical pulses directly into the desired phase of movement [5,6]. FES can improve the upper limb function in post-stroke patients [7,8]. FES also increases the blood flow in the affected cortex area [9]. According to the systematic review [10], FES provides a statistically significant advantage in recovering daily living skills.

Any treatment of post-stroke impairment of the upper limb function requires objective diagnosis and assessment. Special attention should be paid to the active extension of the wrist joint since this movement is necessary to perform grips [11,12] and essential for daily life activities [13,14]. In addition, active wrist extension is a predictor of upper limb functional recovery [15]. As of now, clinical assessment scales and questionnaires remain the main tools for diagnosing upper limb function, although they have been rightly criticized for low reliability and high subjectivity [16,17]. Objective diagnostic methods are being actively studied by the scientific community [18].

One of the objective methods for upper limb movement analysis is video analysis [19,20]. It is highly accurate yet costly and requires a special laboratory setting and considerable time to capture and process data [17].

Electromyography (EMG) is another tool that can be used for objective assessment. Using EMG, Hwang I.S. et al. [21] found that the level of synkinesis in the upper limb after stroke correlates with its functionality. Although being an important tool, EMG provides information only about the electrical activity of muscles but not on the movement kinematics.

Goniometry is a valid method to measure the range of motion, including in the joints of the upper and lower extremities [22]. As of now, however, manual ROM measurements with a goniometer should be regarded as outdated [23]. The method can give information only about the amplitude of wrist motion but not on the process of motion. Inertial measurement units (IMUs) are new-generation devices [24,25]. This technology has achieved accuracy comparable to that of motion capture systems [26]. IMU systems have a number of advantages and can be used outside of special laboratory settings [17].

There are also other methods for objective assessment of upper limb functions, such as electrogoniometry and videoradiography. The first one, however, is technically outdated, and the second one is costly and implies exposure to ionizing radiation [27,28].

The main problem is that, so far, there is no single universal method for instrumental assessment of wrist function, and the existing methods are poorly reproducible [29]. When developing such a method, one should take into account the specifics of muscle functioning, e.g., the fact that a muscle can demonstrate maximum strength from a position of maximum tension [30]. The development of a universal objective method that is affordable to use in routine clinical practice could significantly facilitate objective assessment of functional status.

The purpose of the study was to develop a test for objective assessment of wrist flexion–extension function and to apply the test to evaluate the efficacy of conventional rehabilitation therapy, alone and in combination with FES, in post-stroke patients.

## 2. Materials and Methods

### 2.1. Study Groups

The study was conducted in 2022–2023 at the Biomechanics Laboratory of the Center of Brain Research and Neurotechnologies. The study enrolled a total of 55 subjects, including 15 healthy volunteers without musculoskeletal injuries and diseases (healthy group). The healthy group consisted of 10 females and 5 males, with a mean age of 26.5 ± 3.5 yrs (range: 23 to 33 yrs). Forty subjects after cerebral stroke were divided into two groups. The patient group that received FES in addition to conventional therapy (the FES group) included 16 males and 4 females, with a mean age of 59.7 ± 12 yrs (range: 35–77 yrs). The patient group that received conventional therapy only (the control group) included 13 male and 7 female subjects, with a mean age of 58.3 ± 11.6 yrs (range: 35–77 yrs). One patient withdrew from the study due to a positive Sars-Cov-2 test.

The study was conducted based on the ethical principles outlined in the Declaration of Helsinki; all subjects provided written informed consent. The study was approved by the local ethics committee (No. 11/25-04-22 of 25 April 2022).

Before and after the rehabilitation course, all patients had their upper limb function evaluated using the clinical assessment tools MRC, FMA-UE, and ARAT. In addition, the kinematic parameters of the wrist joints of healthy and paretic limbs were measured using the developed functional test. All subjects were right-handed.

### 2.2. Eligibility Criteria

Inclusion criteria for patients were hemiparetic patients after first-time hemispheric stroke; age up to 80 years; functionally ready for verticalization; with adequate response to orthostatic test; able to keep balance in a sitting position for at least 30 min. Rankin score 3; able to understand and follow simple instructions; stable vegetative and hemodynamic parameters; decrease in muscle strength in the distal part of the upper limb: 1 to 3 points according to MRC; abnormal muscle tone up to 1+ on the modified Ashworth scale; no deep sensibility disorder of the upper limb; no decompensated somatic disorder, no evidence of ischemic changes on the electrocardiogram or heart failure (Killip class II or lower); apart from stroke, no other diseases of the central and peripheral nervous systems accompanied by neurological deficits (sequelae of injuries, tumors, polyneuropathy, etc.); no EEG evidence of epileptic activity; absence of any of the following: orthopedic pathology (joint deformities or contractures, severe pain syndrome, amputated limbs on the paretic side, no operations on paretic upper limb joints with the use of surgical hardware, etc.).

Non-inclusion criteria for patients were an inadequate cardiovascular response to exercise; individual intolerance to percutaneous electrical stimulation; the patient’s refusal of therapy; worsening of neurological and/or somatic status; the presence of implanted pulse generator or artificial pacemaker; the presence of surgical hardware close to the intended area of stimulation; significant pain syndrome in the paretic upper limb at rest or in motion, which prevents exercise; severe cognitive disorders; psychoemotional excitement; signs of hysteria; pseudobulbar syndrome; severe speech disorders; skin lesions; skin diseases of the paretic upper limb; venous thrombosis of lower limbs without signs of recanalization, or arterial thrombosis; parkinsonism and other types of tremor; diagnosed epileptic syndrome.

Exclusion criteria were non-compliance with the protocol, adverse events, and the development of complications or side effects during the study.

### 2.3. Instrumental Technique for Function Assessment

The wrist joint function was assessed using our developed functional test, which consisted of two subtests: wrist joint extension from the neutral position Wrist-0 and from the maximum flexion position Wrist-Flex. The test used two inertial sensors of the Steadys kit (Neurosoft, Ivanovo, Russia) with fixing elastic straps Velkro, a standard software package for data capture, a desk, and a chair.

**Figure 1:**
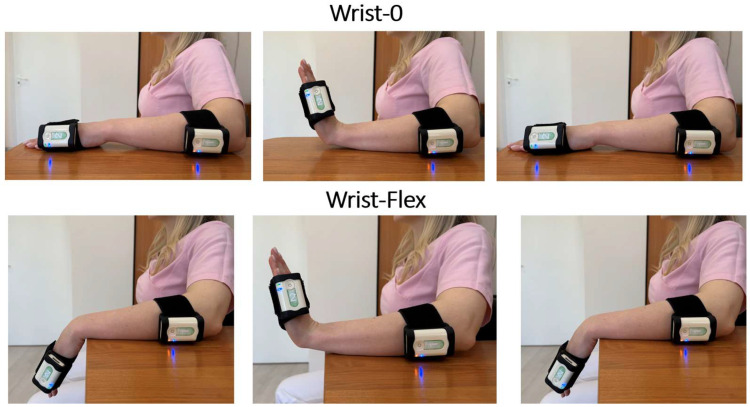
Sensor No. 1 is proximally fixed on the forearm, and sensor No. 2 is on the hand’s edge. The three upper pictures show the Wrist-0 test in progression, from left to right: upper limb position before the start of movement (1), position of maximum wrist extension (2), upper limb position at the end of movement cycle (3). The three lower pictures show the Wrist-Flex test, from left to right: upper limb position before the start (1), position of maximum wrist extension (2), upper limb position at the end of movement cycle (3).

During the assessment, the subject was seated on a chair, leaning against its back, knees bent at 90 degrees, feet firmly touching the floor. The forearm lay on the table, palm down. The sensors were attached to the wrist and the forearm (Figure 1). In the Wrist-0 test, the wrist joint was extended from a neutral position: at the command “Start,” the patient had to perform 3 extension movements of maximum amplitude at an arbitrary pace.

The Wrist-Flex test was performed from a position of complete passive wrist flexion (with the hand hanging freely from the table) and was also performed in full amplitude. At the command “Start,” the patient had to perform the same number of movements as in Wrist-0 at an arbitrary pace.

**Figure 2:**
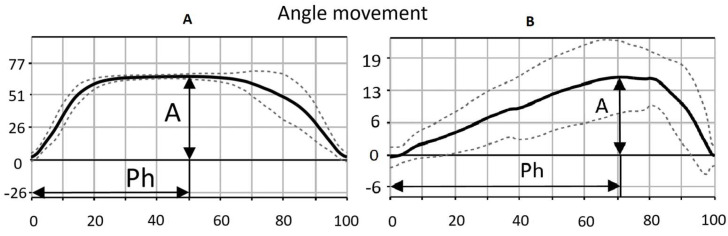
Goniogram (function of angle versus time). The left graph was obtained for a healthy subject, the right one for a post-stroke patient; ***A*** is movement amplitude (in degrees); **Ph** is phase of maximum extension angle (% of full movement cycle). Note that the patient’s range of motion is smaller. In addition, the movement itself is asymmetrical (the maximum amplitude is shifted from the center). Long low-activity flexion and rapid extension under the action of gravity. (**A**) left, (**B**) right panel.

The software automatically processes data, identifies the movement cycles, and presents a mean movement cycle goniogram (Figure 2). The subsequent analysis uses the following parameters: length of movement cycle (s), amplitude (degrees), and phase (% of movement cycle duration).

### 2.4. FES Treatment Session

**Figure 3:**
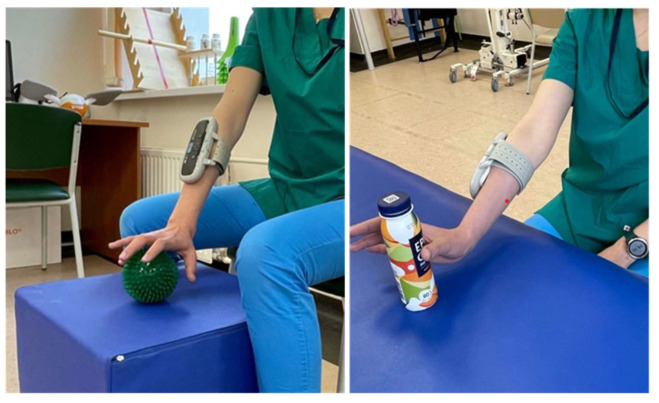
FES session.

FES treatment was performed using an XFT-2003E Hand Rehab System (Shenzhen XFT Medical Limited, Shenzhen, China). The device is equipped with built-in electrodes and a fixing cuff. The device should be placed on the posterior surface of the forearm and attached with the cuff. The inner side of the device (that contacts the skin) has built-in electrodes, which should be positioned in the projection of the extensor muscles of the hand and the fingers. The surface electrodes capture the EMG signal and simultaneously stimulate the same muscles proportionally to the integrated EMG signal received from the same surface electrodes. Before each session, the device was set up based on individual parameters of EMG activity and susceptibility to electrical stimulation. The patient’s task was to grasp and lift spherical and cylindrical objects from horizontal surfaces positioned at various heights (see Figure 3). The stimulation intensity varied, on average, between 20 and 60 μV, the stimulation frequency between 20 and 50 Hz, and the pulse duration, 1 to 2 s. The initial stimulation intensity was set at 20 µV and was gradually increased until the patient started to feel the stimulation but not with discomfort. The frequency was selected in the range of 20–50 Hz, individually for each patient, based on the motor response after preliminary testing. Each patient in the FES group received 10–12 FES sessions in addition to conventional therapy. The stimulation parameters were adjusted on Days 3 and 7 of therapy based on the patient’s subjective feelings and objective assessment of the motor function improvement. Patients in the control group received conventional therapy only. The criteria for discontinuation of FES therapy were as follows: the patient’s complaints of feeling unwell, evident signs of fatigue, and changes in objective parameters indicating the patient’s fatigue. All patients included in the study were subject to daily monitoring by medical personnel. Each FES session was documented in a special log, indicating the date, time, duration, and parameters of stimulation. Any missed sessions were compensated within 48 h.

No adverse events requiring treatment discontinuation were reported in the study. The conventional motor rehabilitation therapy for the upper limb function included 60 min sessions of physical therapy on a Bobath table using manual techniques (Bobath therapy, proprioceptive neuromuscular facilitation, ontogenetic kinesitherapy), mechanotherapy on the Theravital simulator, and individual training with an instructor.

### 2.5. Statistical Analysis

The obtained data were processed by standard methods of descriptive ANOVA using the Statistica 12 software package. The Shapiro–Wilk test was used to check the normality of data distribution. The distribution was not normal (*p* < 0.05). Medians and 25th and 75th percentiles were calculated. The significance of the differences was assessed using Wilcoxon and Mann–Whitney tests, with *p* < 0.05. A comparative analysis of similar parameters of the left and right hands, as well as of the Wrist-0 and Wrist-Flex tests, was performed within groups, between patient groups, and between each group, as well as with a group of healthy individuals. Comparisons were performed between left and right hands, as well as between each patient group and the healthy group. *The effect size was measured using Cohen’s d*.

## 3. Results

This section may be divided by subheadings. It should provide a concise and precise description of the experimental results, their interpretation, as well as the experimental conclusions that can be drawn.

Table 1 presents data obtained using the clinical assessment scales.

The MRC scores of muscle strength showed no significant differences between the two patient groups (FES and Control); there were also no significant changes from baseline after treatment within the groups. The ARAT and FMA-UE scores demonstrated the comparability of the groups at baseline. The post-treatment evaluation showed significant improvements (*p* < 0.05) in both scales within both groups; similar changes were noted in the ICF domain.

Table 2 presents kinematic parameters (amplitude, phase, and duration of movement cycle) for the right and left wrist joints in the group of healthy subjects.

In the healthy group, the movement amplitude in Wrist-0 was significantly smaller than in Wrist-Flex (*p* < 0.05). The phase of maximum angle in Wrist-0 occurred significantly earlier than in Wrist-Flex (*p* < 0.05) for the right arm. The cycle length in Wrist-0 among healthy subjects is significantly shorter than in Wrist-Flex for the left limb. No other significant differences were found.

The kinematic parameters for the paretic limb and their intergroup comparison are shown in Table 3.

In both patient groups before and after treatment, the movement amplitude in Wrist-0 was significantly smaller than in Wrist-Flex (*p* < 0.05). Patients in the FES and control groups demonstrated significant differences from healthy subjects in both Wrist-0 and Wrist-Flex before and after treatment (*p* < 0.05).

The amplitude of wrist movement in the paretic limb significantly improved after treatment in the FES group (*p* < 0.05), whereas no significant improvement was noted in the patient control group.

In the post-treatment FES, the phase of maximum angle in the Wrist-0 test occurred significantly later than in healthy subjects (*p* < 0.05). In other cases, there were no statistically significant differences in the phase of maximum angle between healthy subjects and patients.

In the control group before treatment, the cycle time in the Wrist-0 test was significantly shorter than in the Wrist-Flex test (*p* < 0.05). After treatment, no such differences were found in the group. At the same time, the cycle time in the Wrist-Flex test in the control group after treatment was significantly shorter than before it. In the post-treatment FES group in both tests and in the pre-treatment control group in the Wrist-Flex test, the cycle time was significantly longer than in the healthy group. No other significant differences were found within or between the groups of study subjects.

The results of the functional test on the non-paretic side are presented in Table 4.

The amplitude of wrist movement in the Wrist-Flex test was significantly larger than in the Wrist-0 test in all groups of subjects (*p* < 0.05). Throughout the entire course of therapy, the amplitudes in both tests in the FES group were significantly smaller than in the group of healthy subjects (*p* < 0.05). After treatment, a significant difference was noted between the FES and control groups in both tests (*p* < 0.05). After treatment, the control group showed no differences from the group of healthy subjects in Wrist-Flex.

The phase of maximum angle in the control group in Wrist-0 significantly differed from that in the healthy group (*p* < 0.05), both before and after treatment.

The cycle time in the FES group in the Wrist-Flex test after treatment was significantly longer than in the healthy group; in all other cases throughout the study period, the cycle time did not differ significantly from that in the healthy group. In both patient groups before and after treatment, the Wrist-Flex cycle time was significantly longer than in the Wrist-0 test (*p* < 0.05). After treatment, the cycle time in both tests in the FES group was significantly longer than in the control group.

## 4. Discussion

The two patient groups were fully comparable based on clinical assessments. The MRC scale proved to be insensitive to changes in functional status. The ARAT, FMA-UE, and ICF domain assessments demonstrated significant post-treatment improvements. These scales were more sensitive.

Although the ARAT, FMA-UE, and ICF domains reflect changes, their limited sensitivity does not allow detecting differences between the groups after treatment [16,17,31,32].

The healthy group assessment with the proposed functional test showed no direct significant differences between the left- and right-side parameters. The combined data can, therefore, be used to assess the function of any side. The differences relate only to the subtest parameters. The phase was longer on the right side in the second subtest. The movement cycle in the first subtest was significantly shorter on the left side. These differences are probably due to a stronger arbitrary control of the right hand. The left hand operates mostly automatically, as a result of which the phase of maximum amplitude remained the same in the two subtests, while the movement cycle time was longer in the second one, where the movement amplitude was larger. Thus, this test shows the effect of interhemispheric asymmetry [33,34]. Our data are hardly comparable with those from other relevant studies because our assessment method has not been used before. The closest study by Santos P.S.A. et al. [35] used similar limb positions but focused on tremors. The studies by Costa V. et al. [24] and Wirth M.A. et al. [36] were focused on wrist flexion–extension amplitudes in healthy subjects, and their results are fully consistent with ours.

Analysis of the kinematic parameters of the two patient groups showed their comparability at baseline according to instrumental test results. Patients in both groups significantly differed from healthy subjects in both tests (Wrist-0 and Wrist-Flex). In the Wrist-0 test, the amplitude on the paretic side was significantly smaller than in the Wrist-Flex test, both before and after treatment. After treatment, a significant increase in the amplitude of wrist movement was only noted in the FES group and was probably due to the FES therapy. Similar findings of amplitude reduction in patients versus healthy subjects were obtained in studies by Nam H.S. et al. [37], as well as by Adeel M. et al. [38] and Thies S.B. et al. [29].

In the post-treatment FES group, the phase of maximum angle in both tests occurred later than in the healthy group. In addition, after treatment, the FES group patients had significantly longer cycle times in both tests compared to the healthy subjects. They also showed a delayed phase of maximum amplitude, a known effect described by Shin H. et al. [39]. The delay in the maximum flexion phase may be related to FES, but the nature of the effect requires further study.

In the pre-treatment control group, the cycle time in the Wrist-0 test was significantly shorter than in the Wrist-Flex one. The Wrist-Flex cycle time differed significantly from that in healthy subjects. After the treatment course, no differences were found between the subtests in the control patient group, and the Wrist-Flex cycle time decreased significantly from its pre-treatment value. The post-treatment cycle time did not differ from that in the healthy group in both subtests.

Before treatment, both patient groups demonstrated a reduced amplitude in the healthy limb in both tests compared to healthy subjects. The interhemispheric effect is a known phenomenon in stroke patients and was also noted by other researchers [40,41,42,43]. In the FES group, the amplitude in the non-paretic limb remained significantly smaller than in the control, and the difference persisted after the treatment course. In the control group, however, the post-treatment amplitude in the second subtest did not differ significantly from that in healthy subjects. This effect may be related to the following previously described phenomenon: as the paretic wrist movement amplitude increases, the frequency of the limb spontaneous use increases as well [44].

In both patient groups, the movement cycle time in the non-affected limb in the Wrist-Flex test was longer than in the Wrist-0 test, which was not the case in the group of healthy subjects.

The size effect was assessed using Cohen’s d. The results for all comparisons are given in Table 5.

As one can see, the effect size calculated for the difference in amplitude between the patient group and healthy subjects was within the Huge size effect range, both before and after treatment. Thus, the developed test has shown a significant power for the analysis of movement amplitude. The paretic status of subacute stroke patients could not change significantly over the rather short rehabilitation period. Therefore, the resulting effect sizes after treatment do not differ much from the pre-treatment values.

The effect size for the parameter Phase was Small to Medium, apparently due to the movement asymmetry variability among patients. Unlike healthy subjects, in whom flexion and extension are symmetrical in time, patients show individual asymmetry of flexion–extension movement. Even the very rigid selection criteria applied in the study cannot reduce this variability. In this regard, the movement cycle duration is less variable.

For a medical practitioner, the most interesting question is about the effect of the treatment itself. Based on the most informative parameter—amplitude—the effect size ranges from Medium to Large, but only for the FES group. In the control group, the effect size does not rise above Very small. According to the maximum amplitude phase, the stronger effect is also in the FES group (from Small to Medium).

The comparability of instrumental and clinical tests is a frequently debated issue. For example, the study by McDonnell M.N. et al. [45] showed a high correlation between ARAT and FMA assessments compared to dynamometry data. However, clinical and instrumental tests use different data. Instrumental assessments can provide information that cannot be obtained by the clinical ones. The need for such assessment tools is obvious [29]. Unlike clinical tests, motion analysis systems are able to record all movement data in real-time [26,46]. The analysis of amplitude phase and spatial characteristics of motion is used most often [47].

The proposed functional test is sensitive and can detect not only nuances of recovery progress but also the effect of interhemispheric asymmetry. The test has shown functional differences between patient groups and healthy subjects, proved the comparability of two patient groups in terms of wrist joint function on the paretic side, and detected differences between the two patient groups after a short rehabilitation course. The relatively narrow eligibility criteria for patients allowed us to enroll more homogenous groups, including in terms of wrist function. This enabled us to use fewer patients in this pilot study. The proposed implementation of FES treatment helps to increase the amplitude of wrist movement and improve the wrist function in the paretic limb. At the same time, there is a need to further study its effect on coordination and temporal parameters. The groups’ comparability in kinematic parameters before and after treatment indicates that improvements in the FES group were significant yet not qualitative.

### Study Limitations

This study has a number of limitations. These include a relatively small sample size, which limits the statistical power of the study and its capacity to identify all possible effects. The rather narrow inclusion/non-inclusion criteria were another limiting factor. However, they allowed us to have more homogenous patient groups, given a relatively small sample size. There was poor comparability between the instrumental assessments and the standard clinical scales used in the study. The weight of the applied sensor (40 g) could be critical for the tests. In future studies, it is important to evaluate the long-term effects of FES because the present study focused on its short-term effects.

## 5. Conclusions

The proposed test for the assessment of the wrist flexion–extension function provides objective information; its high sensitivity enables the detection of effects of hemispheric asymmetry in healthy subjects and specific movement impairments in post-stroke patients (decreased amplitude, movement asymmetry, changes in movement duration, decreased function of the healthy side). When used to assess treatment effectiveness, the functional test showed differences between the outcomes of conventional therapy and its combination with FES. The FES-including therapy had a positive effect on the amplitude of wrist movement, while conventional treatment better restored the temporal structure of movement. The study identified typical functional changes in biomechanical gait parameters in subacute stroke patients.

A future study should include broader indications, a longer follow-up monitoring of wrist function, and the long-term outcomes. The study design should provide for an additional group to receive a placebo or dummy FES treatment.

## Figures and Tables

**Table 1:** Clinical assessment scores.

Group/ Parameter	Time Point	Wrist MRC		ARAT	Fugle Maer	ICF d440
		Flexion	Extension			
Control	Before	3.0 [2.0; 3.0]	3.0 [2.0; 3.0]	28.0 [10.75; 32.0]	86.0 [74.75; 95.5]	2.0 [2.0; 3.0]
	After	3.0 [2.0; 3.0]	3.0 [2.0; 3.0]	32.0 [15.25; 42.0] *	96.0 [81.25; 101.5] *	2.0 [1.0; 3.0] *
FES	Before	3.0 [2.0; 3.0]	3.0 [2.0; 3.0]	24.0 [11.0; 32.5]	90.0 [80.02; 99.66]	2.0 [1.5; 3.0]
	After	3.0 [2.0; 3.0]	3.0 [2.2; 3.0]	38.0 [22.5; 40.5] *	102.0 [89.035; 108.83] *	2.0 [1.0; 2.0] *

*—significant difference compared to the same parameter BEFORE (*p* < 0.05).

**Table 2:** Functional test data in healthy subjects.

Test/Parameter	Amplitude (Degrees)		Phase (%)		Cycle (s)	
	Right	Left	Right	Left	Right	Left
Wrist-0	79 [67; 86] *	80 [74; 86] *	41 [38; 45] *	46 [39; 48]	1.78 [1.53; 2.46]	1.85 [1.4; 2.06] *
Wrist-Flex	137 [123; 156]	138 [124; 158]	47 [42; 58]	43 [39; 53]	2.27 [1.7; 2.57]	2.07 [1.74; 2.67]

*—significant difference compared to the same parameter in the Wrist-Flex test (*p* < 0.05).

**Table 3:** Results of the functional test on the paretic side in patient groups.

Group/Parameter	Time Point	Test	Amplitude (Degrees)	Phase (%)	Cycle (s)
Control	Before	Wrist-0	29.0 [8.5; 45.5] *&	51.5 [20.0; 57.5]	2.08 [0.71; 3.02] &
		Wrist-Flex	70.0 [47.5; 87.0] *	51.0 [45.5; 56.5]	2.72 [2.31; 3.725] *
	After	Wrist-0	26.0 [17.5; 53.75] *&	48.0 [38.0; 57.25]	1.96 [1.1525; 2.6925]
		Wrist-Flex	73.0 [44.5; 90.75] *	52.0 [47.5; 56.5]	2.63 [1.9425; 3.12] #
FES	Before	Wrist-0	21.0 [0.0; 27.0] *&	50.0 [0.0; 64.0]	2.135 [0.0; 2.84]
		Wrist-Flex	49.5 [39.0; 65.0] *	48.5 [39.5; 58.0]	2.45 [1.84; 4.015]
	After	Wrist-0	28.5 [21.0; 32.5] #*&	53.5 [47.0; 66.5] *	2.365 [1.94; 3.185]
		Wrist-Flex	59.5 [49.0; 73.5] #*	54.5 [45.0; 59.0]	3.215 [2.11; 4.19] *
Healthy		Wrist-0	79 [68.75; 86.0] &	41 [38.0; 44.75] &	1.78 [1.54; 2.4475]
		Wrist-Flex	137 [123.0; 154.5]	47 [42.25; 56.75]	2.27 [1.79; 2.545]

*—significant difference compared to healthy group (*p* < 0.05); #—significant difference compared to BEFORE (*p* < 0.05); &—significant difference compared to Wrist-Flex (*p* < 0.05).

**Table 4:** Results of the functional test on the non-paretic side in patient groups and in the healthy group.

Group/Parameter	Time Point	Test	Amplitude (Degrees)	Phase (%)	Cycle (s)
Control	Before	Wrist-0	70.0 [65.0; 78.0] *&	48.0 [44.5; 50.5] *	1.93 [1.75; 2.6] &
		Wrist-Flex	125.0 [112.0; 135.0] *	49.0 [45.5; 51.0]	2.415 [2.04; 3.005]
	After	Wrist-0	70.0 [66.25; 78.75] &	47.0 [41.5; 51.5] *	1.67 [1.53; 2.0025] &
		Wrist-Flex	130.0 [120.0; 135.75]	50.0 [46.25; 53.75]	2.05; [1.77; 2.2675]
FES	Before	Wrist-0	64.0 [50.0; 68.5] *&	45.0 [39.25; 51.0]	1.95 [1.8425; 2.1975] &
		Wrist-Flex	114.0 [101.75; 119.75] *	47.0 [42.25; 48.75]	2.53 [1.915; 3.375]
	After	Wrist-0	56.0 [50.5; 67.25] *&@	47.0 [42.75; 49.75]	2.05 [1.75; 2.7475] &@
		Wrist-Flex	110.0 [99.75; 121.75] *@	51.0 [46.25; 55.75]	3.06 [2.09; 3.215] @
Healthy		Wrist-0	79 [68.75; 86.0] &	41 [38.0; 44.75] &	1.78 [1.54; 2.4475]
		Wrist-Flex	137 [123.0; 154.5]	47 [42.25; 56.75]	2.27 [1.79; 2.545]

*—significant difference compared to healthy group (*p* < 0.05); &—significant difference compared to Wrist-Flex (*p* < 0.05); @—significant difference compared to control group (*p* < 0.05).

**Table 5:** The analysis of effect size calculations.

Test	Group	Before and After (Cohen’s d)	Compared to Healthy Group Before Treatment (Cohen’s d)	Compared to Healthy Group After Treatment (Cohen’s d)
Amplitude (degree)				
Wrist-0	FES	0.602 &	−4.754 *	−4.37 *
	Control	0.164	−3.461 *	−2.855 *
Wrist-Flex	FES	0.517	−3.437 *	−3.443 *
	Control	0.101	−2.87 *	−2.569 *
Phase of maximum extension (%)				
Wrist-0	FES	0.381	0.09	−0.486
	Control	0.044	0.097	0.042
Wrist-Flex	FES	0.677 &	0.306	−0.438
	Control	0.083	0.003	−0.079
Cycle duration (t)				
Wrist-0	FES	0.1	−0.284	−0.642
	Control	0.211	0.012	−0.242
Wrist-Flex	FES	0.313	−0.485	−1.27 #
	Control	−0.248	−0.888 #	−0.523

*—Huge size effect; #—Very large size effect; &—Large size effect.

## Data Availability

Data can be obtained from the corresponding author.

## Abbreviations

The following abbreviations are used in this manuscript:

FES	Functional Electrical Stimulation
IMU	Inertial Measurement Unit
MRC	Medical Research Council
FMA-UE	Fugl-Meyer Assessment for Upper Extremity
ARAT	Action Research Arm Test
EEG	Electroencephalography
ICF	International Classification of Functioning
